# Evaluation of the Qvella FAST System and the FAST-PBC cartridge for rapid species identification and antimicrobial resistance testing directly from positive blood cultures

**DOI:** 10.1128/jcm.00569-23

**Published:** 2023-09-28

**Authors:** Issa Sy, Nina Bühler, Sören L. Becker, Philipp Jung

**Affiliations:** 1 Institute of Medical Microbiology and Hygiene, Saarland University, Homburg, Germany; 2 Swiss Tropical and Public Health Institute, Allschwil, Switzerland; 3 University of Basel, Basel, Switzerland; Mayo Clinic, Jacksonville, Florida, USA

**Keywords:** bloodstream infection, blood culture, diagnosis, antimicrobial susceptibility testing, Qvella FAST System, matrix-assisted laser desorption/ionization time-of-flight mass spectrometry (MALDI-TOF MS), MicroScan WalkAway, disk diffusion

## Abstract

Blood culture diagnostics require rapid and accurate identification (ID) of pathogens and antimicrobial susceptibility testing (AST). Standard procedures, involving conventional cultivation on agar plates, may take up to 48 hours or more until AST completion. Recent approaches aim to shorten the processing time of positive blood cultures (PBC). The FAST System is a new technology, capable of purifying and concentrating bacterial/fungal pathogens from positive blood culture media and producing a bacterial suspension called “liquid colony” (LC), which can be further used in downstream analyses (e.g., ID and AST). Here, we evaluated the performance of the FAST System LC generated from PBC in comparison to our routine workflow including ID by matrix-assisted laser desorption/ionization time-of-flight mass spectrometry using Sepsityper, AST by automatized MicroScan WalkAway *plus* and directly inoculated disk diffusion (DD), and MICRONAUT-AM for yeast/fungi. A total of 261 samples were analyzed, of which 86.6% (226/261) were eligible for the comparative ID and AST analyses. In comparison to the reference technique (culture-grown colonies), ID concordance of the FAST System LC and Sepsityper was 150/154 (97.4%) and 123/154 (79.9%), respectively, for Gram positive; 67/70 (95.7%) and 64/70 (91.4%), respectively, for Gram negative. For AST, categorical agreement (CA) of the FAST System LC in comparison to the routine workflow for Gram-positive bacteria was 96.1% and 98.7% for MicroScan and DD, respectively. Similar results were obtained for Gram-negative bacteria with 96.6% and 97.5% of CA for MicroScan and DD, respectively. Taken together, the FAST System LC allowed the laboratory to significantly reduce the time to obtain correct ID and AST (automated MicroScan) results 1 day earlier and represents a promising tool to expedite the processing of PBC.

## INTRODUCTION

Bloodstream infections (BSIs) encompass a large variety of pathogens such as bacteria or fungi causing high rates of mortality and morbidity (1 – 3). Ineffective treatment of BSI due to delayed pathogen identification (ID) and late antimicrobial susceptibility testing (AST) can lead to adverse outcomes (4). Sepsis, defined as an organ dysfunction caused by a dysregulated host response to infection, is considered a major threat and one of the most important causes of health loss and deaths (4, 5). Previous studies estimated around 49 million cases and 11 million deaths per year, which would correspond to 20% of all deaths in the world (5 – 8). In this context, the rapid identification of pathogens followed by AST to enable appropriate antimicrobial therapy is crucial for effective management of septic patients. Since the standard method used in clinical laboratories is based on traditional culture methods and requires at least 24 hours for ID and 48 hours for final AST results, the development of more rapid methods for pathogen ID from positive blood culture (PBC) and subsequent resistance testing is of great interest. To this end, several alternative methods have been developed, which employ molecular detection methods on PBC, such as Biofire FilmArray (3, 9, 10), LightCycler SeptiFast (11), Genmark ePlex (12), Verigene Blood Culture panels (13), Accelerate Pheno system (14), and matrix-assisted laser desorption/ionization time-of-flight mass spectrometry (MALDI-TOF MS) with specific kits (3, 9, 15). In this study, we evaluated the performance of the recently released FAST System (Qvella Corporation, Richmond Hill, Canada)-generated liquid colony (LC) in comparison to our routine workflow, including Sepsityper for rapid MALDI-TOF-based pathogen ID, AST using MicroScan WalkAway *plus*, and directly inoculated disk diffusion (DD).

## MATERIALS AND METHODS

### Sample collection and study design

Between March and November 2022, a total of 261 prospective blood culture samples in different types of BD BACTEC blood culture bottles (Plus Aerobic, Plus Anaerobic, Lytic Anaerobic, Mycosis IC/F, and Peds Plus) (Becton Dickson, Heidelberg, Germany) were collected on different wards at Saarland University Medical Center in Homburg, southwest Germany. After being flagged positive in the BD BACTEC FX System (Becton Dickson), each sample was subjected to Gram staining and further processed using two procedures, which were performed in parallel: (i) the standard routine workflow and (ii) the FAST System workflow.

#### Inclusion/exclusion criteria

All positive blood culture samples obtained during routine diagnostics were included for the comparative assessment, except for the following exclusion criteria: post-mortem blood culture, polymicrobial blood culture (i.e., if different morphologies were seen on Gram staining, samples were not subjected to Qvella FAST System; if culture-grown colonies after 18–24 hours of incubation showed previously unexpected polymicrobial growth, these samples were retrospectively excluded), processing of the blood culture ≥16 hours after being flagged positive, incorrect labeling, and failed runs (cartridge processing errors or system failures).

#### Routine workflow

Each PBC sample was subjected, using the Sepsityper Kit accordingly to the manufacturer’s recommendations, to the Bruker BDAL database using MALDI Biotyper version 3.0 for MALDI-TOF MS ID (Bruker Daltonics, Bremen, Germany). Next, directly inoculated DD was performed using the interpretative recommendations put forth by the European Committee on Antimicrobial Susceptibility Testing (EUCAST) protocol version 12.0, 2022 (16), in order to obtain a preliminary AST result within 18 hours. In brief, 125 ± 5 µL aliquots of positive blood were directly inoculated on Mueller-Hinton or Mueller-Hinton fastidious agar plates (BD Biosciences, Franklin Lakes, NJ, USA), followed by the addition of appropriate antibiotic disks or epsilometry (E-test) gradient strips according to the manufacturer’s protocol (Liofilchem, Italy) and incubated at 35 ± 1°C.

In parallel, the reference method using agar plate subculture was conducted: colonies grown overnight on trypticase soy agar plates supplemented with 5% sheep blood, McConkey agar plates, Columbia agar plates (for anaerobic bacteria), and Sabouraud glucose agar plates (for fungi) (all plates produced by BD Biosciences) were analyzed by MALDI-TOF MS for species ID. Then, agar plate colonies were subjected to automatized AST employing broth microdilution method using the MicroScan WalkAway *plus* System (Beckman Coulter, Germany), with POS MIC33 panels (for Gram-positive bacteria; except for streptococci) and NEG MIC1 panels (for Gram-negative bacteria), according to the manufacturer’s guidelines. If fungal species had been identified, antifungal susceptibility testing (AFST) using MICRONAUT-AM antifungal agent MIC plates (Bruker Daltonics, Germany) was performed.

#### FAST System workflow

The principle of the FAST System is based on the isolation and concentration of microbial cells directly from PBC using single-use FAST PBC Prep cartridges (Qvella Corporation, Canada). For sample preparation, 2-mL aliquots were drawn from PBC bottles. After a run of 24 (for one cartridge) or 38 (for two cartridges) minutes, the bacterial suspension called “liquid colony (LC)” was retrieved and further subjected to downstream analyses such as MALDI-TOF MS ID and AST. For the MALDI ID, 1 µL of the LC was spotted on the MALDI target plate in duplicate. After being dried, each spot was recovered with 1 µL of 70% (vol/vol) formic acid followed by the addition of 1 µL of saturated α-cyano-4-hydroxy-cinnamic acid matrix solution (Bruker Daltonics, Germany). For the AST, both manual (disk diffusion and/or E-tests) and automated ASTs were performed using a bacterial suspension of 0.5 McFarland prepared from the LC. Similar to the routine procedure, the automated AST method was performed using the MicroScan WalkAway *plus* System (Beckman Coulter, Germany), employing POS MIC33 panels (for Gram-positive bacteria; except for streptococci) and NEG MIC1 panels (for Gram-negative bacteria).

### Comparative analysis

ID and AST (or AFST) results obtained with the two aforementioned workflows (routine workflow and FAST workflow) were compared for concordance. In addition, refer to the description of Inclusion/exclusion criteria, given above.

#### MALDI-TOF ID comparison

MALDI-ID obtained via Sepsityper or via the FAST System LC was compared to MALDI-ID obtained by culture-grown colonies as reference method. As two spots were measured for each sample, only the MALDI ID with the highest identification log score value was considered during the comparison (log score values ≥2 were considered as reliable species level ID, and score values ≥1.7, as probable species ID). Inconclusive IDs correspond to identification results with a score value below the threshold or “no peak found.” Discrepant IDs correspond to identifications of different species for the same sample using two methods.

#### AST (or AFST) comparison

Results from DD (including *E*-tests) and automated AST (MicroScan WalkAway) (or AFST if fungal species were identified) obtained with the FAST System LC were compared to AST (or AFST) results obtained with the routine workflow. Categorical agreement (CA) was employed for the interpretation of the different AST results: “susceptible” (S), “resistant” (R), and “susceptible, increased exposure” (I) following the breakpoints put forth by EUCAST version 12.0 (16). Of note, AST results (i.e., MIC or diameter zone) in the area of technical uncertainty occurred in rare cases and were interpreted as given in Supplemental file S1**-** Table S1. Discrepancies were evaluated based on error types including minor errors (minE: results that are categorized increased [I] in one workflow but susceptible [S] or resistant [R] in the other), major errors (majE: results that are resistant [R] in the FAST System LC workflow but susceptible [S] in the routine workflow), and very major errors (VmajE: results that are susceptible [S] in the FAST System LC workflow, but resistant [R] in the routine workflow). Of note, as preliminary AST (i.e., directly inoculated DD) was compared with the DD results obtained from the FAST System workflow, isolates in which discrepancies (VmajE, majE, and minE) occurred were retested by performing standard DD from grown colonies obtained after reinoculation of cryo-cultures in order to clarify these discrepancies. The percentage of CA is calculated by dividing the number of categorical matches by the total number of antibiotics tested with the reference method × 100. The rate of VmajE was calculated by dividing the number of VmajE by the total number of resistant bacteria tested with the reference method × 100. The rate of majE was calculated by dividing the number of majE by the total number of susceptible bacteria tested with the reference method × 100. The rate of minE was calculated by dividing the number of minE by the total number of antibiotics tested with the reference method × 100.

### Time to results

To estimate the rapidity of the FAST System workflow compared to the routine workflow, the time to ID and AST results was calculated by measuring the necessary time to obtain conclusive ID and AST results for both workflows. For the FAST System, the time was measured with a run of two cartridges simultaneously. Statistical tests were performed using analysis of variance (ANOVA), and a *P*-value <0.05 was considered as significant.

## RESULTS

### Study sample characteristics

Thirty-five out of 261 (13.4%) samples were excluded from the analysis, including 28 (10.7%) polymicrobial PBCs that were not detected by the initial Gram staining and 7 (2.7%) failed runs corresponding to cartridge processing errors or software failures. The remaining 226 (86.6%) samples were eligible for comparison.

### MALDI-TOF ID comparison

When being compared to the reference technique (culture-grown colonies), MALDI-TOF ID following the FAST workflow showed 96.9% (219/226) concordance, two discrepant IDs, and five inconclusive IDs (no peaks found or score value under the threshold). Sepsityper showed 82.74% (187/226) concordance, 1 discrepant ID, and 38 inconclusive IDs. The 219 samples correctly identified with the FAST System comprised 150 Gram-positive bacteria, i.e., 120/123 (97.6%) staphylococci, 19/20 (95%) enterococci, and 9/9 (100%) streptococci, as well as 1/1 (100%) *Micrococcus luteus* and 1/1 (100%) *Paenibacillus timonensis* (Table 1). The 69 remaining samples revealed 67 Gram-negative bacteria [59/61 (96.7%) Enterobacterales, with 33/33 (100%) *Escherichia coli* and 11/11 (100%) *Klebsiella pneumoniae* being the most represented species, 6/7 (85.7%) *Pseudomonas* spp., 1/1 (100%) *Bacteroides fragilis*, and 1/1 (100%) *Elizabethkingia anophelis*) (Table 2), and two yeasts both identified as *Candida glabrata*. Overall, the most common species were *Staphylococcus epidermidis* (*n* = 64) and *Escherichia coli* (*n* = 33). The two yeast species were successfully identified as *C. glabrata* following the FAST workflow (with MALDI score values of 1.86 and 1.93), while they were not successfully identified when the routine workflow (Sepsityper) was applied. Average MALDI scores of both workflows reached reliable values, comparable to those obtained for culture-grown colonies. For Gram-positive bacteria, we obtained scores of 2.00, 2.05, and 2.19, respectively, for FAST System, Sepsityper, and culture-grown colonies. While for Gram-negative bacteria, scores were 2.16, 2.12, and 2.24, respectively, for FAST System LC, Sepsityper, and culture-grown colonies. Likewise, log score values ≥2.00 were obtained for all groups (staphylococci, enterococci, Enterobacterales, etc.) with all three methods (Tables 1 and 2). Nevertheless, a few ID errors corresponding to inconclusive ID (i.e., no MALDI peaks found or MALDI score value below the threshold) and/or discrepant ID were observed. For Gram-positive bacteria, ID errors occurred in 4/154 (2.6%), 31/154 (20.1%), and 6/154 (3.9%) instances for FAST System, Sepsityper, and culture-grown colonies, respectively (Table 1). For Gram-negative bacteria, ID errors were observed in 3/70 (4.3%), 6/70 (8.6%), and 4/70 (5.7%) cases when using the FAST System, Sepsityper, and culture-grown colonies, respectively (Table 2). As compared to the reference (culture-grown colonies), MALDI ID using the LC from the FAST System revealed no discrepancies for Gram-positive bacteria. For Gram-negative bacteria, two discrepancies were noticed in the Enterobacterales group (*K. pneumoniae* were obtained instead of *K. variicola*) (Table 2).

**TABLE 1:** Identification of Gram-positive bacteria isolated from PBCs by MALDI-TOF MS using three different methods

Gram-positive bacteria			
	Culture-grown colonies (%)^*c*^	Sepsityper (%)	FAST System (%)
Staphylococci	119/123 (96.7%) 2.17^*b*^	99/123 (80.5%) 2.07^*b*^	120/123 (97.6%) 2.01^*b*^
*Staphylococcus epidermidis*	63/65 (96.9%)	52/65 (80%)	64/65 (98.5%)
*Staphylococcus aureus*	28/29 (96.6%)	24/29 (82.8%)	29/29 (100%)
*Staphylococcus hominis*	11/11 (100%)	9/11 (81.8%)	10/11 (90.9%)
*Staphylococcus haemolyticus*	7/8 (87.5%)	5/8 (62.5%)	7/8 (87.5%)
*Staphylococcus capitis*	6/6 (100%)	6/6 (100%)	6/6 (100%)
*Staphylococcus saccharolyticus*	2/2 (100%)	1/2 (50%)	2/2 (100%)
*Staphylococcus caprae*	1/1 (100%)	1/1 (100%)	1/1 (100%)
*Staphylococcus lugdunensis*	1/1 (100%)	1/1 (100%)	1/1 (100%)
Inconclusive ID	4/123 (3.3%)	24/123 (19.5%)	3/123 (2.4%)
Discrepant ID	NA^*a*^	0/123 (0%)	0/123 (0%)
Enterococci	18/20 (90%) 2.34^*b*^	16/20 (80%) 2.00^*b*^	19/20 (95%) 2.12^*b*^
*Enterococcus faecium*	10/11 (90.9%)	9/11 (81.8%)	10/11 (90.9%)
*Enterococcus faecalis*	7/8 (87.5%)	6/8 (75%)	8/8 (100%)
*Enterococcus casseliflavus*	1/1 (100%)	1/1 (100%)	1/1 (100%)
Inconclusive ID	2/20 (10%)	4/20 (20%)	1/20 (5%)
Discrepant ID	NA^*a*^	0/20 (0%)	0/20 (0%)
Streptococci	9/9 (100%) 2.14^*b*^	7/9 (77.8%) 2.02^*b*^	9/9 (100%) 1.92^*b*^
*Streptococcus mitis*	3/3 (100%)	2/3 (66.7%)	3/3 (100%)
*Streptococcus oralis*	2/2 (100%)	2/2 (100%)	2/2 (100%)
*Streptococcus dysgalactiae*	1/1 (100%)	1/1 (100%)	1/1 (100%)
*Streptococcus gallolyticus*	1/1 (100%)	1/1 (100%)	1/1 (100%)
*Streptococcus gordonii*	1/1 (100%)	1/1 (100%)	1/1 (100%)
*Streptococcus parasanguinis*	1/1 (100%)	0/1 (0%)	1/1 (100%)
Inconclusive ID	0/9 (0%)	1/9 (11.1%)	0/9 (0%)
Discrepant ID	NA^*a*^	1/9 (11.1%)	0/9 (0%)
Other Gram positives	2/2 (100%) 2.18^*b*^	1/2 (50%) 2.26^*b*^	2/2 (100%) 2.12^*b*^
*Micrococcus luteus*	1/1 (100%)	0/1 (0%)	1/1 (100%)
*Paenibacillus timonensis*	1/1 (100%)	1/1 (100%)	1/1 (100%)
Inconclusive ID	0/2 (0%)	1/2 (50%)	0/2 (0%)
Discrepant ID	NA^*a*^	0/2 (0%)	0/2 (0%)
Total Gram positive	148/154 (96.1%) 2.19^*b*^	123/154 (79.9%) 2.05^*b*^	150/154 (97.4%)2.00^*b*^

^*a*^ NA, not applicable.

^*b*^ Average MALDI score.

^*c*^ Reference technique.

**TABLE 2:** Identification of Gram-negative bacteria isolated from PBCs by MALDI-TOF MS using three different methods

Gram-negative bacteria			
	Culture-grown colonies (%)^*c*^	Sepsityper (%)	FAST System (%)
Enterobacterales	57/61 (93.4%) 2.25^*b*^	57/61 (93.4%) 2.14^*b*^	59/61 (96.7%) 2.19^*b*^
*Escherichia coli*	32/33 (97%)	32/33 (97%)	33/33 (100%)
*Klebsiella pneumoniae*	10/11 (90.9%)	11/11 (100%)	11/11 (100%)
*Klebsiella variicola*	4/5 (80%)	4/5 (80%)	3/5 (60%)
*Enterobacter cloacae complex*	4/4 (100%)	3/4 (75%)	4/4 (100%)
*Citrobacter koseri*	2/2 (100%)	2/2 (100%)	2/2 (100%)
*Proteus mirabilis*	2/2 (100%)	2/2 (100%)	2/2 100%)
*Klebsiella oxytoca*	1/2 (50%)	1/2 (50%)	2/2 100%)
*Morganella morganii*	1/1 (100%)	1/1 (100%)	1/1 100%)
*Serratia marcescens*	1/1 (100%)	1/1 (100%)	1/1 100%)
Inconclusive ID	4/61 (6.6%)	4/61 (6.6%)	0/61 (0%)
Discrepant ID	NA^*a*^	0/61 (0%)	2/61 (3.3%)
*Pseudomonas* spp.	7/7 (100%) 2.24^*b*^	6/7 (85.7%) 2.08^*b*^	6/7 (85.7%) 2.14^*b*^
*Pseudomonas aeruginosa*	5/5 (100%)	4/5 (80%)	4/5 (80%)
*Pseudomonas montelii*	2/2 (100%)	2/2 (100%)	1/1
*Pseudomonas* sp.	NA^*a*^	NA^*a*^	1/1
Inconclusive ID	0/7 (3.3%)	1/7 (14.3%)	1/7 (14.3%)
Discrepant ID	NA^*a*^	0/7 (0%)	0/7 (0%)
Other Gram negatives	2/2 (100%) 2.19^*b*^	1/2 (50%) 2.03^*b*^	2/2 (100%) 2.07^*b*^
*Bacteroides fragilis*	1/1 (100%)	0/1 (0%)	1/1 (100%)
*Elizabethkingia anophelis*	1/1 (100%)	1/1 (100%)	1/1 (100%)
Inconclusive ID	0/2 (0%)	1/2 (50%)	0/2 (0%)
Discrepant ID	NA^*a*^	0/2 (0%)	0/2 (0%)
Total Gram negative	66/70 (94.3%); 2.24^*b*^	64/70 (91.4%); 2.12^*b*^	67/70 (95.7%); 2.16^*b*^

^*a*^ NA, not applicable.

^*b*^ Average MALDI score.

^*c*^ Reference technique.

### AST (or AFST) comparison

Manual and automated AST (or AFST) results obtained with the two workflows (routine and FAST) were compared using categorical agreement based on EUCAST guidelines version 12.

Automated AST results are presented in Table 3 (Gram positive) and Table 4 (Gram negative) with a total of 129 Gram-positive and 58 Gram-negative samples analyzed, comprising 115 staphylococci, 14 enterococci, 54 Enterobacterales, and 4 *Pseudomonas* spp. Of note, AST of 17 LC (five staphylococci and five enterococci, five Enterobacterales, and two *Pseudomonas* spp.) were not analyzed due to insufficient biomass to prepare a suspension of 0.5 McFarland or a failed run of the MicroScan. The rates of CA, VmajE, majE, and minE were, respectively, 1119/1165 (96.1%), 8/265 (3%), 16/824 (1.9%), and 22/1165 (1.9%) for staphylococci and 110/114 (96.5%), 2/22 (9.1%), 2/83 (2.4%), and 0/114 (0%) for enterococci (Table 3). For Enterobacterales, rates of CA, VmajE, majE, and minE were 964/991 (97.3%), 16/152 (10.5%), 4/788 (0.5%), and 7/991 (0.7%), respectively, and 29/37 (78.4%), 0/1 (0%), 1/7 (14.3%), and 7/37 (18.9%) for *Pseudomonas* spp. (Table 4).

**TABLE 3:** Automated AST results from MicroScan WalkAway of Gram-positive bacteria (staphylococci and enterococci) following the FAST System workflow as compared to our routine workflow^*a*^

Antibiotic	CA (%)	VmajE (%)	MajE (%)	MinE (%)
Staphylococci (*N* = 115)				
Clindamycin	105/115 (91.3)	2/40 (5)	3/70 (4.3)	5/115 (4.3)
Daptomycin	86/87 (98.9)	0/0 (NA)	1/87 (1.1)	0/87 (0)
Flucloxacillin	109/115 (94.8)	3/63 (4.8)	3/52 (5.8)	0/115 (0)
Fosfomycin	113/115 (98.3)	1/17 (5.9)	1/98 (1)	0/115 (0)
Gentamicin	108/114 ((94.7)	2/50 (4)	4/64 (6.3)	0/114 (0)
Levofloxacin	113/115 (98.3)	0/60 (0)	0/1 (0)	2/115 (1.7)
Linezolid	86/87 (98.9)	0/0 (NA)	1/87 (1.1)	0/87 (0)
Rifampicin	114/115 (99.1)	0/17 (0)	0/97 (0)	1/115 (0.9)
Teicoplanin	74/74 (100)	0/0 (NA)	0/74 (0)	0/74 (0)
Co-trimoxazole	99/115 (86.1)	0/18 (0)	2/81 (2.5)	14/115 (12.2)
Vancomycin	112/113 (99.1)	0/0 (NA)	1/113 (0.9)	0/113 (0)
Total	1119/1165 (96.1)	8/265 (3)	16/824 (1.9)	22/1165 (1.9)
Enterococci (*N* = 14)				
Ampicillin	14/14 (100)	0/5 (0)	0/9 (0)	0/14 (0)
Ciprofloxacin	14/14 (100)	0/6 (0)	0/8 (0)	0/14 (0)
Gentamicin	13/14 (92.9)	1/4 (25)	0/10 (0)	0/14 (0)
Imipenem	9/9 (100)	0/0 (NA)	0/0 (NA)	0/9 (0)
Levofloxacin	14/14 (100)	0/6 (0)	0/8 (0)	0/14 (0)
Linezolid	13/14 (92.9)	0/0 (NA)	1/14 (7.1)	0/14 (0)
Nitrofurantoin	8/8 (100)	0/0 (NA)	0/8 (0)	0/8 (0)
Teicoplanin	13/13 (100)	0/0 (NA)	0/13 (0)	0/13 (0)
Vancomycin	12/14 (85.7)	1/1 (100)	1/13 (7.7)	0/114 (0)
Total	110/114 (96.5)	2/22 (9.1)	2/83 (2.4)	0/114 (0)

^*a*^ CA, categorical agreement; VmajE, number of very major errors encountered; majE, number of major errors encountered; minE, number of minor errors encountered; NA, not applicable.

**TABLE 4:** Automated AST results from MicroScan WalkAway of Gram-negative bacteria (Enterobacterales and *Pseudomonas* spp.) following the FAST System workflow as compared to our routine workflow^*a*^

Antibiotic	CA (%)	VmajE (%)	MajE (%)	MinE (%)
Enterobacterales (*N* = 54)				
Ampicillin	50/54 (92.6)	2/40 (5)	2/14 (14.3)	0/54 (0)
Amoxicillin-clavulanic acid	53/54 (98.1)	0/18 (0)	1/36 (2.8)	0/54 (0)
Piperacillin-tazobactam	52/54 (96.3)	2/7 (28.6)	0/42 (0)	0/54 (0)
Pivmecillinam	17/17 (100)	0/0 (NA)	0/17 (0)	0/17 (0)
Cefuroxime	48/53 (90.6)	2/12 (16.7)	0/0 (NA)	3/53 (5.7)
Cefotaxime	50/53 (94.3)	2/8 (25)	0/44 (0)	1/53 (1.9)
Ceftazidime	52/54 (96.3)	1/8 (12.5)	0/45 (0)	1/54 (1.9)
Cefepime	53/54 (98.1)	1/7 (14.3)	0/47 (0)	0/54 (0)
Ceftazidime-avibactam	6/6 (100)	0/0 (NA)	0/6 (0)	0/6 (0)
Ertapenem	54/54 (100)	0/1 (0)	0/53 (0)	0/54 (0)
Imipenem	51/51 (100)	0/1 (0)	0/50 (0)	0/51 (0)
Meropenem	54/54 (100)	0/1 (0)	0/53 (0)	0/54 (0)
Gentamicin	54/54 (100)	0/7 (0)	0/47 (0)	0/54 (0)
Tobramycin	53/54 (98.1)	1/8 (12.5)	0/46 (0)	0/54 (0)
Amikacin	54/54 (100)	0/0 (NA)	0/54 (0)	0/54 (0)
Ciprofloxacin	52/54 (96.3)	0/7 (0)	0/44 (0)	2/54 (3.7)
Levofloxacin	54/54 (100)	0/7 (0)	0/47 (0)	0/54 (0)
Co-trimoxazole	54/54 (100)	0/13 (0)	0/41 (0)	0/54 (0)
Fosfomycin	48/54 (88.9)	5/6 (83.3)	1/48 (2.1)	0/54 (0)
Nitrofurantoin	32/32 (100)	0/1 (0)	0/31 (0)	0/32 (0)
Trimethoprim	23/23 (100)	0/0 (NA)	0/23 (0)	0/23 (0)
Total	964/991 (97.3)	16/152 (10.5)	4/788 (0.5)	7/991 (0.7)
Pseudomonas spp. (*N* = 4)				
Piperacillin-tazobactam	4/4 (100)	0/0 (NA)	0/0 (NA)	0/4 (0)
Ceftazidime	4/4 (100)	0/0 (NA)	0/0 (NA)	0/4 (0)
Cefepime	4/4 (100)	0/0 (NA)	0/0 (NA)	0/4 (0)
Ertapenem	1/1 (100)	0/1 (0)	0/0 (NA)	0/1 (0)
Imipenem	3/4 (75)	0/0 (NA)	0/0 (NA)	1/4 (25)
Meropenem	4/4 (100)	0/0 (NA)	0/4 (0)	0/4 (0)
Tobramycin	3/4 (75)	0/0 (NA)	1/1 (100)	0/4 (0)
Amikacin	4/4 (100)	0/0 (NA)	0/0 (NA)	0/4 (0)
Ciprofloxacin	1/4 (25)	0/0 (NA)	0/1 (0)	3/4 (75)
Levofloxacin	1/4 (25)	0/0 (NA)	0/1 (0)	3/4 (75)
Total	29/37 (78.4)	0/1 (0)	1/7 (14.3)	7/37 (18.9)

^*a*^ CA, categorical agreement; VmajE, number of very major errors encountered; majE, number of major errors encountered; minE, number of minor errors encountered; NA, not applicable.

For DD, a total of 201 bacteria were analyzed, including 135 Gram-positive bacteria (113 staphylococci, 16 enterococci, 5 streptococci, and 1 *Micrococcus luteus*) and 66 Gram-negative bacteria (59 Enterobacterales, 5 *Pseudomonas* spp., 1 *B. fragilis*, and 1 *E. anophelis*). The percentages of CA, VmajE, majE, and minE were 434/436 (99.5%), 1/75 (1.3%), 1/361 (0.3%), and 0/436 (0%), respectively, for staphylococci; 85/89 (95.5%), 4/23 (17.4%), 0/59 (0%), and 0/89 (0%) for enterococci; 23/25 (92%), 2/3 (66.7%), 0/22 (0%), and 1/25 (4%) for streptococci; and 4/4 (100%), 0/1 (0%), 0/3 (0%), and 0/4 (0%) for *M. luteus* (Table 5). In Gram-negative bacteria, CA, VmajE, majE, and minE were 338/347 (97.4%), 1/26 (3.8%), 3/315 (0.9%), and 5/347 (1.4%), respectively, for Enterobacterales; 48/49 (98%), 0/2 (0%), 0/16 (0%), and 1/49 (2%), respectively, for *Pseudomonas* spp.; 6/6 (100%), 0/4 (0%), 0/2 (0), and 0/6 (0%), respectively, for other Gram-negative bacteria (i.e*.*, *B. fragilis* and *E. anophelis*) (Table 6).

**TABLE 5:** Manual AST results from DD of Gram-positive bacteria (staphylococci, enterococci, streptococci, and *M. luteus*) following the FAST System workflow as compared to our routine workflow (DD)^*a*^

Antibiotic	CA (%)	VmajE (%)	MajE (%)	MinE (%)
Staphylococci (*N* = 113)				
Cefoxitin	108/108 (100)	0/59 (0)	0/49 (0)	0/108 (0)
Flucloxacillin	1/1 (100)	0/1 (0)	0/0 (NA)	0/1 (0)
Linezolid	112/112 (100)	0/2 (0)	0/110 (0)	0/112 (0)
Rifampicin	108/110 (98.2)	1/13 (7.7)	1/97 (1)	0/110 (0)
Vancomycin	105/105 (100)	0/0 (NA)	0/105 (0)	0/105 (0)
Total	434/436 (99.5)	1/75 (1.3)	1/361 (0.3)	0/436 (0)
Enterococci (*N* = 16)				
Ampicillin	15/15 (100)	0/8 (0)	0/7 (0)	0/15 (0)
Gentamicin	13/14 (92.9)	1/3 (33.3)	0/11 (0)	0/14 (0)
Imipenem	15/15 (100)	0/8 (0)	0/0 (NA)	0/15 (0)
Linezolid	14/14 (100)	0/0 (NA)	0/14 (0)	0/14 (0)
Tigecycline	14/15 (93.3)	1/1 (100)	0/14 (0)	0/15 (0)
Vancomycin	14/16 (87.5)	2/3 (66.7)	0/13 (0)	0/16 (0)
Total	85/89 (95.5)	4/23 (17.4)	0/59 (0)	0/89 (0)
Streptococci (*N* = 5)				
Clarithromycin	2/2 (100)	0/1 (0)	0/1 (0)	0/2 (0)
Clindamycin	4/4 (100)	0/0 (NA)	0/4 (0)	0/4 (0)
Erythromycin	3/5 (60)	2/2 (100)	0/3 (0)	0/5 (0)
Penicillin	5/5 (100)	0/0 (NA)	0/5 (0)	0/5 (0)
Co-trimoxazole	4/4 (100)	0/0 (NA)	0/4 (0)	1/4 (25)
Vancomycin	5/5 (100)	0/0 (NA)	0/5 (0)	0/5 (0)
Total	23/25 (92)	2/3 (66.7)	0/22 (0)	1/25 (4)
Micrococcus luteus (*N* = 1)				
Cefuroxime	1/1 (100)	0/0 (NA)	0/1 (0)	0/1 (0)
Ciprofloxacin	1/1 (100)	0/1 (0)	0/0 (NA)	0/1 (0)
Linezolid	1/1 (100)	0/0 (NA)	0/1 (0)	0/1 (0)
Meropenem	1/1 (100)	0/0 (NA)	0/1 (0)	0/1 (0)
Total	4/4 (100)	0/1 (0)	0/3 (0)	0/4 (0)

^*a*^ CA, categorical agreement; VmajE, number of very major errors encountered; majE, number of major errors encountered; minE, number of minor errors encountered; NA, not applicable.

**TABLE 6:** Manual AST results from DD of Gram-negative bacteria (Enterobacterales, *Pseudomonas* spp., *B. fragilis*, and *E. anophelis*) following the FAST System workflow as compared to our routine workflow (DD)^*a*^

Antibiotic	CA (%)	VmajE (%)	MajE (%)	MinE (%)
Enterobacterales (*N* = 59)				
Piperacillin-tazobactam	56/58 (96.6)	1/8 (12.5)	1/50 (2)	0/59 (0)
Cefotaxime	57/58 (98.3)	0/7 (0)	1/51 (2)	0/58 (0)
Ceftazidime	56/59 (94.9)	0/4 (0)	0/52 (0)	3/59 (5.1)
Ceftazidime-avibactam	56/57 (98.2)	0/0 (NA)	1/57 (1.8)	0/57 (0)
Meropenem	58/58 (100)	0/0 (NA)	0/57 (0)	0/58 (0)
Ciprofloxacin	55/57 (96.5)	0/7 (0)	0/48 (0)	2/57 (3.5)
Total	338/347 (97.4)	1/26 (3.8)	3/315 (0.9)	5/347 (1.4)
Pseudomonas spp. (*N* = 5)				
Piperacillin-tazobactam	5/5 (100)	0/0 (NA)	0/0 (NA)	0/5 (0)
Cefiderocol	2/2 (100)	0/0 (NA)	0/2 (0)	0/2 (0)
Ceftazidime	5/5 (100)	0/0 (NA)	0/0 (NA)	0/5 (0)
Cefepime	5/5 (100)	0/0 (NA)	0/0 (NA)	0/5 (0)
Ceftazidime-avibactam	2/2 (100)	0/0 (NA)	0/2 (0)	0/2 (0)
Ceftolozane-tazobactam	2/2 (100)	0/0 (NA)	0/2 (0)	0/2 (0)
Imipenem	5/5 (100)	0/1 (0)	0/0 (NA)	0/5 (0)
Meropenem	5/5 (100)	0/1 (0)	0/2 (0)	0/5 (0)
Tobramycin	4/4 (100)	0/0 (NA)	0/4 (0)	0/4 (0)
Amikacin	4/4 (100)	0/0 (NA)	0/4 (0)	0/4 (0)
Ciprofloxacin	5/6 (83.3)	0/0 (NA)	0/0 (NA)	1/6 (16.7)
Levofloxacin	4/4 (100)	0/0 (NA)	0/0 (NA)	0/4 (0)
Total	48/49 (98)	0/2 (0)	0/16 (0)	1/49 (2)
Other Gram-negatives (B. fragilis [*n* = 1], and E. anophelis [*n* = 1])				
Penicillin	1/1 (100)	0/1 (0)	0/0 (NA)	0/1 (0)
Piperacillin-tazobactam	2/2 (100)	0/1 (0)	0/1 (0)	0/2 (0)
Clindamycin	1/1 (100)	0/0 (NA)	0/1 (0)	0/1 (0)
Meropenem	1/1 (100)	0/1 (0)	0/0 (NA)	0/1 (0)
Ciprofloxacin	1/1 (100)	0/1 (0)	0/0 (NA)	0/1 (0)
Total	6/6 (100)	0/4 (0)	0/2 (0)	0/6 (0)

^*a*^ CA, categorical agreement; VmajE, number of very major errors encountered; majE, number of major errors encountered; minE, number of minor errors encountered; NA, not applicable.

Furthermore, a complete overview of AST results is shown in Supplemental file S2-Table S4. Herein, for Gram-positive bacteria, the CA of the FAST LC in comparison to the routine procedure reached 96.1% and 98.4% for MicroScan and DD results, respectively. Similar results were obtained for Gram-negative bacteria, with CA of 96.6% and 97.5% for MicroScan and DD, respectively (Supplemental file S2**-**Table S4).

However, a few discrepancies were also encountered. For MicroScan AST results of Gram-positive pathogens, a total of 50 errors were recorded. Most of the errors were minE (*n* = 22), detected mainly in *S. epidermidis* isolates with co-trimoxazole (13 discrepancies detected in 63 *S. epidermidis* tested) (Supplemental file S2**-** Table S1), followed by majE (*n* = 18) found mainly in *S. epidermidis* (three discrepancies with flucloxacillin, two with gentamicin, and two with co-trimoxazole detected in 63 *S. epidermidis* tested), and VmajE (*n* = 10) where gentamicin (*n* = 3) and flucloxacillin (*n* = 3) showed most discrepancies (Supplemental file S2-Table S1). When Gram-negative pathogens were tested, the majority of the 35 discrepancies were VmajE (*n* = 16) found mostly in coliform bacilli with fosfomycin (*n* = 5) being the most common discrepant substance, followed by minE (*n* = 14) and majE (*n* = 5) (Supplemental file S2-Table S1). Considering these results and to the best of our knowledge, our study is the first one combining the FAST LC with MicroScan WalkAway to assess automated AST.

For manual AST (DD), first, 53 discrepancies (21 for Gram positive and 32 for Gram negative) were found between the FAST System LC and our routine workflow based on direct inoculation DD of PBC, which was analyzed after 18 hours. Next, to clarify these errors, all isolates presenting these errors were re-analyzed by performing standard DD inoculated from grown colonies obtained after reinoculation of cryo-cultures on blood agar plates. It is important to highlight that 71.7% (38/53) of discrepant AST results were resolved, including 22 in Gram-negative bacteria and 16 in Gram-positive bacteria (Supplemental file S2-Table S2). Hence, the remaining discrepancies were for Gram-positive bacteria, seven VmajE mainly found in enterococci (*n* = 4) with gentamicin, tigecycline, and vancomycin, one majE in *S. epidermidis* with rifampicin, and one minE in *S. mitis* with co-trimoxazole (Supplemental file S2-Table S3). While for Gram-negative bacteria, 10 discrepancies were found: one VmajE in *E. coli* with piperacillin-tazobactam; three majE in *E. coli* (*n* = 2) with piperacillin-tazobactam and ceftazidime-avibactam and in *K. variicola* (*n* = 1) with cefotaxime; and 6 minE in *E. coli*, *K. variicola*, *K. pneumoniae*, and *P. aeruginosa* with ceftazidime and ciprofloxacin (Supplemental file S2-Table S3).

In addition, the two yeasts species (*C. glabrata*), which were correctly identified using the FAST System LC, showed 14/16 (87.5%) CA and 1/16 (6.3%) minE found with itraconazole. The only resistant profile found with itraconazole using the reference method showed a discrepancy (1/1 VmajE) when compared to the AST result obtained via the FAST System (Table 7; Supplemental file S2-Table S4).

**TABLE 7:** Micronaut AST results of *C. glabrata* following the FAST System workflow as compared to our routine workflow^*a*^

Antibiotic	CA (%)	VmajE (%)	MajE (%)	MinE (%)
C. glabrata (*N* = 2)				
Amphotericin	2/2 (100)	0/0 (NA)	0/2 (0)	0/2 (0)
5-Fluorocytosin	2/2 (100)	0/0 (NA)	0/2 (0)	0/2 (0)
Fluconazole	2/2 (100)	0/0 (NA)	0/2 (0)	0/2 (0)
Voriconazole	2/2 (100)	0/0 (NA)	0/2 (0)	0/2 (0)
Micafungin	2/2 (100)	0/0 (NA)	0/2 (0)	0/2 (0)
Anidulafungin	2/2 (100)	0/0 (NA)	0/2 (0)	0/2 (0)
Caspofungin	2/2 (100)	0/0 (NA)	0/2 (0)	0/2 (0)
Itraconazole	0/2 (0)	1/1 (100)	0/0 (NA)	1/2 (50)
Total	14/16 (87.5)	1/1 (100)	0/14 (0)	1/16 (6.3)

^*a*^ CA, categorical agreement; VmajE, number of very major errors encountered; majE, number of major errors encountered; minE, number of minor errors encountered; NA, not applicable.

### Time to results

**Fig 1:**
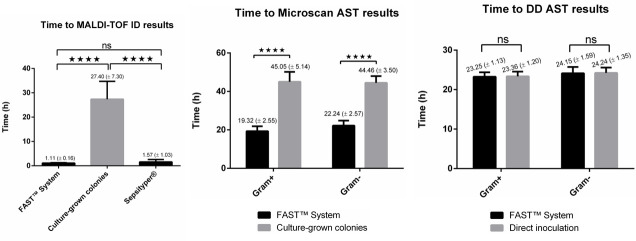
Average time required to obtain ID and AST results (by MicroScan WalkAway *plus* and direct-inoculation DD) using different methods: FAST System, Sepsityper, and culture-grown colonies.

The time to obtain ID and AST results using the FAST System LC was estimated in comparison to our routine workflow. Here, the time required to obtain correct MALDI ID and MicroScan AST results by means of FAST System LC was significantly reduced by 1 day as compared to the routine workflow using culture-grown colonies. Hence, the average time for the FAST System LC to obtain ID results was 1.11 (±0.16) hours. While for the workflow of the reference method using overnight culture in our laboratory, the average time was 27.4 (±7.30) hours. This difference is statistically significant (*P* < 0.05). In contrast, the difference between FAST and Sepsityper is not significant. For MicroScan AST, the average times for Gram-positive pathogens were 19.3 (±2.55) and 45.1 (±5.14) hours for FAST System LC and culture-grown colonies, respectively. Similarly, for Gram-negative pathogens, 22.2 (±2.6) and 44.5 (±3.5) hours were necessary to obtain MicroScan AST results, respectively, for FAST System workflow and culture-grown colonies. Both differences were statistically significant (*P* < 0.05). For DD AST, the turnaround time for both Gram positive and Gram negative was comparable, and there was no significant difference between FAST System and direct inoculation (Fig. 1).

## DISCUSSION

One of the most important objectives in clinical microbiology is to provide a rapid and accurate microbiological diagnosis of BSI. Thus, tools to accelerate PBCs processing are urgently needed. Many of those tools are based on syndromic nucleic acid amplification such as multiplex PCR, which provides good sensitivity but are usually limited to a preselected panel of pathogens. Furthermore, such molecular techniques are limited by the types of organisms and resistance mechanisms included in the panel. In this study, we examined an alternative approach to accelerating culture-based processing. The FAST System generates a LC after an approximative run of 24 minutes by purifying the pathogens directly from the positive blood culture. Here, IDs (by MALDI-TOF MS) and AST (by MicroScan WalkAway *plus* and disk diffusion) results were analyzed and compared to those obtained via a culture-based routine workflow. By using the FAST System LC, correct and accurate ID results were achieved with a concordance of 96.9% as compared to the reference technique (culture-grown colonies). A concordance rate of 82.74% was achieved with Sepsityper. Similar results with Sepsityper were previously reported by Morgenthaler and Kostrzewa with 80% (*n* = 3320) species ID (17). Regarding the performance of the FAST System in terms of ID, Grinberg and colleagues reported an ID concordance of 94% (*n* = 201) using MALDI Biotyper as compared to colonies obtained by subculture (18). Similarly, Verroken and colleagues reported 89.5% (*n* = 266) concordant ID results using the FAST System LC and MALDI Biotyper including 80 blood cultures bottles spiked with multidrug-resistant bacteria (19). Likewise, Kuo and colleagues demonstrated an accurate ID of 94.1% (272/289) using LC generated by the FAST System and MALDI-TOF MS Microflex (20). Fifty-four of the 272 samples corresponded to initially negative blood culture bottles which were seeded with key organisms (e.g., *Streptococcus agalactiae*, *Streptococcus pyogenes*, *Klebsiella* spp., *Proteus mirabilis*, *Enterobacter* spp., *Escherichia coli*., etc.) in order to have a more diverse and larger set of bacteria with highly resistant patterns. All 54 samples (100%) were correctly identified with the LC suspension. Hence, by using combined prospective and spiked data set, they demonstrated that a wide array of clinically relevant Gram-positive and Gram-negative bacteria were identifiable using the LC suspension produced by the FAST System (19, 20).

In our study, FAST-DD led to slightly better results (CA of 98.7% for Gram positive and 97.5% for Gram negative) when compared to directly inoculated DD from PBC, which are part of our routine workflow, than the automated method using FAST-MicroScan (CA of 96.1% for both Gram positive and Gram negative). Comparable CA values were previously reported for both Gram-positive [97.4% (18), 97.7%–100% (19), and 99.5% (20)] and Gram-negative bacteria [98.5% (18), 97.8%–99% (19), and 97.8% (20)] where automated AST systems other than MicroScan WalkAway (i.e., bioMérieux Vitek 2 or BD Phoenix) were utilized. Given these previous studies, our study though is the first to use MicroScan WalkAway demonstrating the compatibility of this automated AST technology with the FAST System. Nevertheless, a few VmajE and majE were reported, which could have a clinical impact on patient treatment, especially for VmajE, where a patient might be treated with antibiotics that are supposed to be sensitive when they are resistant to that treatment. Most VmajE and majE were detected for staphylococcal and Enterobacterales species, which are also the most frequently detected pathogen groups, without any noticeable error accumulation for specific substances tested among those groups (for details refer to Results: AST comparison or Supplemental material S2**-**Tables S1 to S3).

An important limitation of the FAST System is the lacking ability to process polymicrobial PBC. This has to be taken into account, since polymicrobial BSIs are considerably detected with a range of 6%–32% of all diagnosed BSI (21, 22). Another limitation can be seen in the fact that a single instrument can only process two PBC cartridges simultaneously. Therefore, for potential implementation in a routine workflow, one would consider having multiple instruments or selecting urgent samples (e.g., an ICU patient) that should be prioritized for processing. Only one instrument was utilized during the period of this study.

In addition, several limitations of our study are offered for consideration. First, it was a monocentric analysis with a moderate sample size. Second, the number of some pathogens (e.g., yeast) obtained during the investigation was too low to draw meaningful conclusions. Hence, we can simply rely on this limited number of samples, which limits the impact of the described findings. It is important to also mention that the FAST Prep cartridges utilized in this study were designed, primarily, for bacteria only. However, the results obtained with yeasts indicate a potential application to yeast samples, as well. Therefore, it would be interesting to perform additional investigations on a larger number of yeast samples to accurately evaluate the performance of the FAST System LC for yeast ID and AFST.

In summary, the FAST System LC enables a turnaround time reduction of approximately 24 hours for conclusive ID results. In addition, MicroScan AST results could be obtained with a reduction time of 24 hours from PBCs with similar accuracy compared to subculture-based reference techniques. The system connects well with Bruker Biotyper MALDI-TOF MS and MicroScan WalkAway *plus,* as it provides enough biomass to perform ID and inoculate DD and MicroScan AST plates. Our findings and those made in previous studies indicate that the FAST System LC approach is capable of delivering more timely results that can improve the outcomes of patients suffering from BSI.

## SUPPLEMENTAL MATERIAL

Supplemental file S1-Table S1 jcm.00569-23-s0001.pdf AST results in the Area of technical uncertainty (ATU)

Supplemental file S2-Tables S1-S4 jcm.00569-23-s0002.pdf Discrepant AST results and overview of global AST results

ASM does not own the copyrights to Supplemental Material that may be linked to, or accessed through, an article. The authors have granted ASM a non-exclusive, world-wide license to publish the Supplemental Material files. Please contact the corresponding author directly for reuse.
