# Supplementary material for: Evaluation of the Qvella FAST System and the FAST-PBC cartridge for rapid species identification and antimicrobial resistance testing directly from positive blood cultures
Source: J Clin Microbiol. 2023 Sep 28;61(10):e00569-23. doi: 10.1128/jcm.00569-23 (PMC10595056; doi:10.1128/jcm.00569-23)
Supplement: Supplemental file S1-Table S1 — AST results in the Area of technical uncertainty (ATU) [file jcm.00569-23-s0001.pdf]

Supplemental file S1-Table S1. Automated (MicroScan) and manual (DD) AST: results in the area of technical uncertainty (ATU)

	Sample ID	Species	MIC (mg/L)	Interpretation	ATU (Y/N)	Interpretation Comparative Analyses
**Automated AST (MicroScan): results in the area of technical uncertainty (ATU)**						
Enterobacterales						
Piperacillin-tazobactam: EUCAST ATU = 16 mg/L (version 2022)						
	Sample ID	Species	MIC (mg/L)	Interpretation	ATU (Y/N)	Interpretation Comparative Analyses
FAST™ LC	171	*E. coli*	16	R	Yes	CA
Reference	196	*K. pneumoniae*	16	R	Yes	VmajE
Ciprofloxacin: EUCAST ATU = 0.5 mg/L (version 2022)						
	ID Sample	Species	MIC	Interpretation	ATU (Y/N)	Interpretation Comparative Analyses
FAST™ LC	8	*K. pneumoniae*	0.5	I	Yes	CA
	49	*P. aeruginosa*	0.5	I	Yes	minE
	255	*E. coli*	0.5	I	Yes	minE
Reference	8	*K. pneumoniae*	0.5	I	Yes	CA
**Manual AST (disk diffusion): results in the area of technical uncertainty (ATU)**						
Enterobacterales						
Piperacillin-tazobactam: EUCAST ATU = 19 mm (version 2022)						
	Sample ID	Species	Zone diameter (mm)	Interpretation	ATU (Y/N)	Interpretation Comparative Analyses
FAST™ LC	18	*K. pneumoniae*	19	R	Yes	CA
	171	*E. coli*	19	R	Yes	CA
	212	*E. coli*	19	R	Yes	CA
Reference	43	*E. coli*	19	R	Yes	VmajE
Ciprofloxacin: EUCAST ATU = 22-24 mm (version 2022)						
	Sample ID	Species	Zone diameter (mm)	Interpretation	ATU (Y/N)	Interpretation Comparative Analyses
FAST™ LC	73	*E. coli*	24	I	Yes	minE
	210	*E. coli*	24	I	Yes	CA
Reference	8	*K. pneumoniae*	23	I	Yes	minE
	210	*E. coli*	23	I	Yes	CA
Staphylococci						
Cefoxitin: EUCAST ATU = 27 mm (version 2022)						
	Sample ID	Species	Zone diameter (mm)	Interpretation	ATU (Y/N)	Interpretation Comparative Analyses
FAST™ LC	33	*S. epidermidis*	27	S	Yes	CA
Reference	208	*S. lugdunensis*	27	S	Yes	CA
