# Supplementary material for: Evaluation of the Qvella FAST System and the FAST-PBC cartridge for rapid species identification and antimicrobial resistance testing directly from positive blood cultures
Source: J Clin Microbiol. 2023 Sep 28;61(10):e00569-23. doi: 10.1128/jcm.00569-23 (PMC10595056; doi:10.1128/jcm.00569-23)
Supplement: Supplemental file S2-Tables S1-S4 — Discrepant AST results and overview of global AST results [file jcm.00569-23-s0002.pdf]

Table S1. Discrepant AST results (VmajE, MajE, and MinE) encountered in Gram-positive and Gram-negative bacteria using the FAST™ System and automated MicroScan WalkAway.

Discrepant automated AST (MicroScan) of Gram-positive bacteria					
**Antibiotic**	**Species**	**N**	**VmajE**	**MajE**	**MinE**
Clindamycin	*S. hominis*	10	1	NA	NA
	S. aureus	29	1	NA	1
	S. epidermidis	63	NA	1	4
	S. haemolyticus	7	NA	2	NA
Daptomycin	*S. haemolyticus*	7	NA	1	NA
Flucloxacillin	*S. hominis*	10	1	NA	NA
	S. hominis	10	1	NA	NA
	S. epidermidis	63	1	3	NA
Fosfomycin	*S. epidermidis*	63	1	NA	NA
	S. hominis	10	NA	1	NA
Gentamicin	*S. aureus*	29	2	2	NA
	E. faecium	5	1	NA	NA
	S. epidermidis	63	NA	2	NA
Vancomycin	*E. casseliflavus*	1	1	NA	NA
	S. haemolyticus	7	NA	1	NA
	E. faecalis	8	NA	1	NA
Linezolid	*S. haemolyticus*	7	NA	1	NA
	E. faecalis	8	NA	1	NA
Co-trimoxazole	*S. epidermidis*	63	NA	2	13
	S. hominis	10	NA	NA	1
Levofloxacin	*S. aureus*	29	NA	NA	1
	S. epidermidis	63	NA	NA	1
Rifampicin	*S. aureus*	29	NA	NA	1
		**Total**	**10**	**18**	**22**
**Discrepant automated AST (MicroScan) of Gram-negative bacteria**					
**Antibiotic**	**Species**	**N**	**VmajE**	**MajE**	**MinE**
Ampicillin	*E. coli*	31	1	1	NA
	E. cloacae complex	1	1	NA	NA
	K. oxytoca	2	NA	1	NA
Amoxicillin-clavulanic acid	*K. pneumoniae*	11	NA	1	NA
Piperacillin-tazobactam	*S. marcescens*	1	1	NA	NA
	K. pneumoniae	11	1	NA	NA
Cefuroxime	*K. variicola*	3	2	NA	NA
	E. coli	31	NA	NA	2
	K. pneumoniae	11	NA	NA	1
Cefotaxime	*S. marcescens*	1	1	NA	NA
	K. pneumoniae	11	1	NA	NA
	K. variicola	3	NA	NA	1
Ceftazidime	*S. marcescens*	1	1	NA	NA
	E. coli	31	NA	NA	1
Cefepime	*K. variicola*	3	1	NA	NA
Imipenem	*P. aeruginosa*	3	NA	NA	1
Tobramycin	*S. marcescens*	1	1	NA	NA
	P. monteilii	1	NA	1	NA
Ciprofloxacin	*E. coli*	31	NA	NA	2
	P. aeruginosa	3	NA	NA	2
	P. monteilii	1	NA	NA	1
Levofloxacin	*P. aeruginosa*	3	NA	NA	2
	P. monteilii	1	NA	NA	1

Fosfomycin	*K. pneumoniae*	11	2	NA	NA
	K. variicola	3	1	NA	NA
	P. mirabilis	2	1	NA	NA
	E. cloacae complex	1	1	NA	NA
	S. marcescens	1	NA	1	NA
		**Total**	**16**	**5**	**14**

N, number of species tested; VmajE, number of very major errors encountered; majE, number of major errors encountered; minE, number of minor errors encountered; NA, not applicable.

Table S2. Discrepancies (VmajE, majE, and minE) encountered with directly inoculated DD and repeatedly tested with standard DD.

**Gram-positive**							
ID	Organism	Substance	FAST™ DD	directly-inoculated DD	Discrepant: yes/no	standard DD	Solved: yes/no
41	*S. epidermidis*	Cefoxitin	S	R	yes	S	yes
59	*S. epidermidis*	Rifampicin	S	R	yes	R	no
85	*S. aureus*	Linezolid	S	R	yes	S	yes
		Rifampicin	S	I	yes	S	yes
81	*E. faecalis*	Vancomycin	S	R	yes	R	no
87	*E. faecium*	Gentamicin	S	R	yes	S	yes
96	*S. epidermidis*	Cefoxitin	S	R	yes	S	yes
118	*E. faecium*	Gentamicin	S	R	yes	S	yes
126	*S. hominis*	Rifampicin	S	I	yes	S	yes
130	*E. faecium*	Linezolid	S	R	yes	S	yes
		Tigecycline	S	R	yes	S	yes
136	*S. hominis*	Cefoxitin	S	R	yes	S	yes
151	*E. faecalis*	Tigecycline	S	R	yes	S	yes
156	*E. faecium*	Linezolid	S	R	yes	S	yes
		Tigecycline	S	R	yes	S	yes
157	*E. casseliflavus*	Vancomycin	R	S	yes	R	yes
166	*E. faecium*	Gentamicin	S	R	yes	R	no
218	*E. faecalis*	Tigecycline,	S	R	yes	S	yes
		Vancomycin	S	R	yes	S	yes
238	*E. faecium*	Tigecycline	S	R	yes	R	no
		Vancomycin	S	R	yes	R	no
							total 21: 16 yes, 5 no
**Gram-negative**							
ID	Organism	Substance	FAST™ DD	directly-inoculated DD	Discrepant: yes/no	standard DD	Solved: yes/no
8	*K. pneumoniae*	Ciprofloxacin	S	I	yes	I	no
16	*E. coli*	Ceftazidime	S	I	yes	S	yes
40	*E. coli*	Piperacillin-Tazobactam	S	R	yes	S	yes
43	*E. coli*	Piperacillin-Tazobactam	S	R	yes	R	no
49	*P. aeruginosa*	Ciprofloxacin	R	I	yes	I	no

73	*E. coli*	Piperacillin-Tazobactam	R	S	yes	S	no
		Ciprofloxacin	I	S	yes	S	no
74	*K. variicola*	Ceftazidime	S	I	yes	I	no
		Cefotaxime	R	S	yes	S	no
75	*K. pneumoniae*	Ceftazidime	S	I	yes	S	yes
86	*S. marcescens*	Ceftazidime	S	I	yes	S	yes
95	*E. coli*	Ciprofloxacin	S	I	yes	S	yes
109	*K. pneumoniae*	Meropenem	I	R	yes	S	yes
131	*Enterobacter cloacae*	Piperacillin-Tazobactam	S	I	yes	S	yes
		Ceftazidime	S	R	yes	S	yes
		Cefotaxime	S	R	yes	S	yes
139	*E. coli*	Ceftazidime	S	I	yes	I	no
173	*K. pneumoniae*	Piperacillin-Tazobactam	S	R	yes	S	yes
175	*K. pneumoniae*	Ceftazidime	S	I	yes	S	yes
		Meropenem	S	I	yes	S	yes
		Ciprofloxacin	S	I	yes	S	yes
		Piperacillin-Tazobactam	S	R	yes	S	yes
185	*K. pneumoniae*	Meropenem	S	I	yes	S	yes
		Ciprofloxacin	S	I	yes	S	yes
198	*E. cloacae complex*	Ceftazidime	S	I	yes	S	yes
244	*E. coli*	Ceftazidime	S	I	yes	S	yes
245	*P. monteilii*	Meropenem	I	R	yes	I	yes
		Levofloxacin	I	R	yes	I	yes
255	*E. coli*	Ciprofloxacin	S	I	yes	S	yes
256	*E. coli*	Ceftazidime	I	R	yes	R	no
261	*E. coli*	Ceftazidime-Avibactam	R	S	yes	S	no
		Ciprofloxacin	S	I	yes	S	yes
							total 32: 22 yes, 10 no

S, susceptible; R, resistant; I, increased susceptible.

Table S3. Discrepant AST results (VmajE, MajE, and MinE) encountered in Gram-positive and Gram-negative bacteria using the FAST™ System and manual AST (DD).

**Discrepant manual AST (DD) of Gram-positive bacteria**					
**Antibiotic**	**Species**	**N**	**VmajE**	**MajE**	**MinE**
Gentamicin	*E. faecium*	8	1	NA	NA
Rifampicin	*S. epidermidis*	61	1	1	NA
Tigecycline	*E. faecium*	8	1	NA	NA
Vancomycin	*E. faecalis*	7	1	NA	NA
	E. faecium	8	1	NA	NA
Erythromycin	*S. dysgalactiae*	1	1	NA	NA
	S. mitis	3	1	NA	NA
Co-trimoxazole	*S. mitis*	3	NA	NA	1
		**Total**	**7**	**1**	**1**

Discrepant manual AST (DD) of Gram-negative bacteria					
**Antibiotic**	**Species**	**N**	**VmajE**	**MajE**	**MinE**
Piperacillin-tazobactam	*E. coli*	33	1	1	NA
Cefotaxime	*K. variicola*	3	NA	1	NA
Ceftazidime-avibactam	*E. coli*	33	NA	1	NA
Ceftazidime	*E. coli*	33	NA	NA	2
	K. variicola	3	NA	NA	1
Ciprofloxacin	*K. pneumoniae*	11	NA	NA	1
	P. aeruginosa	3	NA	NA	1
	E. coli	33	NA	NA	1
		**Total**	**1**	**3**	**6**

N, number of species tested; VmajE, number of very major errors encountered; majE, number of major errors encountered; minE, number of minor errors encountered; NA, not applicable.

Table S4. Categorical agreement of AST results from MicroScan WalkAway, disk diffusion, and MICRONAUT-AM following the FAST™ workflow in comparison to our routine workflow.

**Gram-positive**															
	**Routine - MicroScan**				FAST™ LC **MicroScan**		**%**		**Routine - DD**				FAST™ LC **DD**		**%**
	**S**	**I**	**R**	**Total**	**CA**	1229	96.1		**S**	**I**	**R**	**Total**	**CA**	546	98.4
**S**	889	7	10	906	**VmajE**	10	3.3	**S**	444	0	7	451	**VmajE**	7	6.9
**I**	0	63	13	76	**majE**	18	2	**I**	0	7	0	7	**majE**	1	0.2
**R**	18	2	277	297	**minE**	22	1.7	**R**	1	1	95	97	**minE**	1	0.2
**Total**	907	72	300	1279				**Total**	445	8	102				
**Gram-negative**															
	**Routine - MicroScan**				FAST™ LC **MicroScan**		**%**		**Routine - DD**				FAST™ LC **DD**		**%**
	**S**	**I**	**R**	**Total**	**CA**	993	96.6		**S**	**I**	**R**	**Total**	**CA**	391	97.5
**S**	797	7	16	820	**VmajE**	16	10.2	**S**	329	3	1	333	**VmajE**	1	3
**I**	1	59	4	64	**majE**	5	0.6	**I**	1	31	1	33	**majE**	3	0.9
**R**	5	2	137	144	**minE**	14	1.4	**R**	3	1	31	35	**minE**	6	1.5
Total	803	68	157	1028				**Total**	333	35	33	401			
**Yeast (*C. glabrata*)**															

	Routine MICRONAUT-AM				FAST™ LC MICRONAUT-AM		%
	S	I	R	Total	CA	14	87.5
S	14	0	1	15	VmajE	1	100
I	0	0	0	0	majE	0	0.0
R	0	1	0	1	minE	1	6.3
Total	14	1	1	16			
